# Voluntary alcohol consumption is increased in female, but not male, oxytocin receptor knockout mice

**DOI:** 10.1002/brb3.1749

**Published:** 2020-07-14

**Authors:** Karla M. Rodriguez, Brittany L. Smith, Heather K. Caldwell

**Affiliations:** ^1^ School of Biomedical Sciences and the Brain Health Research Institute Kent State University Kent OH USA; ^2^ Laboratory of Neuroendocrinology and Behavior Department of Biological Sciences Kent State University Kent OH USA; ^3^ Department of Pharmacology & Systems Physiology University of Cincinnati Cincinnati OH USA

**Keywords:** forced swim test, knockout mice, oxytocin, oxytocin receptor, voluntary alcohol consumption

## Abstract

**Introduction:**

The oxytocin (Oxt) system, while typically associated with the neural regulation of social behaviors, also plays a role in an individual's vulnerability to develop alcohol use disorders (AUD). In humans, changes to the Oxt system, due to early life experience and/or genetic mutations, are associated with increased vulnerability to AUD. While a considerable amount is known about Oxt's role in AUD in males, less is known or understood, about how Oxt may affect AUD in females, likely due to many clinical and preclinical studies of AUD not directly considering sex as a biological variable. This is unfortunate given that females are more vulnerable to the effects of alcohol and have increased alcohol consumption, as compared to males. Therefore, in the current study we wanted to determine whether genetic disruption of the Oxt receptor (Oxtr), that is, Oxtr knockout (−/−) mice, affected stress‐induced alcohol consumption in males and females. We hypothesized that genetic disruption of the Oxtr would result in increased stress‐induced alcohol consumption in both males and females compared to wild‐type (+/+) controls. Though, we predicted that these disruptions might be greater in female Oxtr −/− mice.

**Methods:**

To test this hypothesis, a two‐bottle preference test was utilized along with the forced swim test (FST), and pre‐ and poststress alcohol consumption and preference measured within each sex (males and females were run separately). As a follow‐up experiment, a taste preference test, to control for possible genotypic differences in taste, was also performed.

**Results:**

In males, we found no significant genotypic differences in alcohol consumption or preference. However, in females, we found that genetic disruption of the Oxtr resulted in a greater consumption of alcohol both pre‐ and poststress compared to controls.

**Conclusion:**

These data suggest that in females, disruptions in Oxt signaling may contribute to increased vulnerability to alcohol‐associated addiction.

## Graphical abstract

Our study is the first to investigate alcohol consumption using Oxtr −/− mice. In females, but not males, we found that genetic disruption of the Oxtr resulted in a greater consumption of alcohol both pre‐ and poststress compared to controls. These data suggest that in females, disruptions in Oxt signaling may contribute to increased vulnerability to alcohol‐associated disorders.

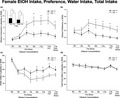

## 1 INTRODUCTION

The central oxytocin (Oxt) system, while primarily known for its role in the modulation of social behaviors, is also implicated in addiction‐associated behaviors (Caldwell, 2018; Caldwell & Albers, 2016; Kovacs, Sarnyai, & Szabo, 1998). This is perhaps not surprising, as many individuals that abuse drugs also have impaired social interactions (Schuckit, 2009). There is also evidence that the Oxt system can be altered by chronic drug use (Bowen & Neumann, 2017; Kovacs et al., 1998; Lee & Weerts, 2016). For instance, in the case of alcohol, chronic exposure results in the degeneration of Oxt‐containing magnocellular neurons in the hypothalamus (Silva, Madeira, Ruela, & Paula‐Barbosa, 2002; Sivukhina, Dolzhikov, Morozov Iu, Jirikowski, & Grinevich, 2006; Stevenson et al., 2017). This loss of oxytocinergic function, in addition to the stress‐associated components of addiction, is widely associated with increased drug‐seeking behavior, neurocognitive deficits, and changes in sociability (McGregor & Bowen, 2012; Sinha, 2008). Beyond these use‐associated changes to the endogenous Oxt system, there is also evidence that the Oxt system may contribute to an individual's predisposition to develop an addictive disorder.

In humans, single nucleotide polymorphisms (SNPs) of the oxytocin receptor (Oxtr) are associated with development of alcohol use disorders (AUD). In children, Vaht et al. (2016) found a linkage between the OXTR rs53576 polymorphism and alcohol use. Specifically, males homozygous for the A allele (thought to be associated with less efficient oxytocinergic functioning), are more frequent alcohol consumers at ages 15 and 18 and more likely to have alcohol abuse or addiction issues later in life. In females, no association between this polymorphism and alcohol use was identified. Though, males and females that are AA homozygous, who also reported less favorable relations with their teachers, are more likely to develop AUD later in life (Vaht, Kurrikoff, Laas, Veidebaum, & Harro, 2016). While the linkage between the Oxt system and AUD appears weak in females, it is of note that the Vaht et al., 2016 study is the only one that has evaluated the effects of Oxt SNPs on alcohol consumption in females. So, the relationship between the Oxt system and AUD in females is largely still unknown. In another study, conducted only in males, polymorphisms in the OXTR are associated with exposure to substance misuse intervention, peer substance abuse, and adolescent alcohol use during the 9th grade (Cleveland et al., 2018). Although further research is needed, these data support the idea that a disrupted Oxt system may predispose an individual to AUD.

While animal models of AUD and alcohol consumption, similar to the human studies, have largely utilized male subjects, the data from preclinical models generally support clinical work. Specifically, they suggest that a functional Oxt system may help protect an individual from developing AUD, whereas a dysfunctional Oxt system may increase an individual's vulnerability for developing AUD. Rodent studies have shown that administration of Oxt reduces alcohol consumption, alcohol withdrawal symptoms, and alcohol‐induced side effects, including blocking of tolerance and hypothermic effects in mice and rats (Tirelli, Jodogne, & Legros, 1992). Oxt also improves the tolerance for the myorelaxant and akinesia effects of alcohol (Bowen, Carson, Spiro, Arnold, & McGregor, 2011; Bowen et al., 2015; Jodogne, Tirelli, Klingbiel, & Legros, 1991; King et al., 2017; Tirelli et al., 1992). Beyond the ligand, a functional Oxtr seems to be critical for the protective effects of Oxt; specifically, its expression in the nucleus accumbens (NAcc). Overexpression of the Oxtr in the NAcc, delivered via lentiviral vector, reduces voluntary alcohol consumption (Bahi, Al Mansouri, & Al Maamari, 2016) and intraperitoneal (IP) pretreatment with an Oxtr antagonist blocks Oxt's ability to reduce binge‐like drinking in male mice (King et al., 2017). Preclinical studies in prairie voles have found that systemic release of Oxt, facilitated by social interactions with a same‐sex familiar partner, decreases the probability of alcohol relapse (Hostetler & Ryabinin, 2014). These data suggest that changes in the Oxt system, either the receptor or the ligand, may affect an individual's vulnerability to abuse alcohol.

While the above data strongly support the assertion that the Oxt system can affect alcohol use and dependency, as has been mentioned previously, most of this work has been performed in males, due in part to men having higher prevalence rates of AUD compared to women (Abuse & Administration, 2016). Females, however, should not be ignored because they tend to have a different alcohol metabolism, increased blood alcohol levels, and greater neurotoxic effects compared to males (Anker & Carroll, 2011). In preclinical studies, female rodents often self‐administer greater volumes of alcohol and maintain a longer preference for alcohol compared to males (Almeida et al., 1998; Juarez & Barrios de Tomasi, 1999; Lancaster, Brown, Coker, Elliott, & Wren, 1996; Sneddon, White, & Radke, 2019; Vetter‐O'Hagen, Varlinskaya, & Spear, 2009). There are also notable sex differences in the brain's Oxt system. Generally speaking, females tend to have higher expression levels of Oxt and Oxtr as compared to males (Dumais, Bredewold, Mayer, & Veenema, 2013; Uhl‐Bronner, Waltisperger, Martinez‐Lorenzana, Condes Lara, & Freund‐Mercier, 2005). Given that males and females differ in their physiological and behavioral responses to alcohol and that there are sex differences in the Oxt system (Anker & Carroll, 2011; Heather K. Caldwell, 2018), it is reasonable to predict that the Oxt system may have differing effects on alcohol consumption in males and females. It is within this framework that the current study was performed.

The goal of this study was to understand how a dysfunctional Oxt system affected alcohol consumption in both males and females, rather than directly evaluating sex differences. Taking into account previous research that suggests Oxt is able to offer some protection against excessive alcohol consumption, through its actions on the Oxtr (Bahi et al., 2016; King et al., 2017; Schuckit & Hesselbrock, 1994), and female vulnerability to alcohol abuse (Anker & Carroll, 2011), we sought to investigate how genetic disruption of the Oxtr affected stress‐induced alcohol consumption in female and male Oxtr knockout (−/−) mice. We *hypothesized* that female and male Oxtr −/− mice would have increased voluntary alcohol consumption compared to wild‐type littermates. We predicted that these disruptions might be greater in females due to noted differences in the Oxt system. As work focused on disruption of the Oxt system and alcohol consumption is limited, this study was designed to shed some light on Oxt‐dependent differences in alcohol consumption following a physical stressor in both males and females.

## 2 MATERIALS AND METHODS

### 2.1 Animals

Adult male and female Oxtr −/− and wild‐type (+/+) mice [males: Oxtr +/+ *N* = 8, Oxtr −/− *N* = 11 (9–26 weeks old), females: Oxtr +/+ *N* = 9, Oxtr −/− *N* = 10 (9–20 weeks old)] were generated from heterozygous breeding pairs and maintained on a 12:12 light‐dark cycle (lights on 0200h). These mice are C57BL/6J‐backcrossed as described in Macbeth, Lee, Edds, and Young (2009). Animals were individually housed in standard mouse cages (7.5W x 11.5L x 5H inches) and given ad libitum access to standard mouse chow and water. Female mice were freely cycling for this study. Genotyping was performed as described previously by H. J. Lee, Caldwell, Macbeth, Tolu, and Young (2008) using purified DNA collected by tail biopsy after weaning at PND 21. All procedures were conducted in accordance with protocols approved by the Kent State University Animal Care and Use Committee.

### 2.2 Voluntary Ethanol (EtOH) Consumption

All animals were given continuous access to two 50‐ml plastic centrifuge tubes with a 2.5” sipper tube, one containing drinking tap water and the other containing EtOH (v/v). The concentration of EtOH was sequentially increased in the following manner: 7 days at 0%, 7 days at 3%, 7 days at 5%, 7 days at 7%, 7 days at 9%, and 7 days of 11%. Following the 7‐day exposure to 11% EtOH, mice were physically stressed once per day for three consecutive days using the forced swim test (FST) (described below). Mice were then maintained at 11% EtOH for 14 days. Thus, prestress consumption of EtOH and poststress consumption of EtOH were measured; this experimental design is described in Caldwell et al. (2006). Throughout the experiment, bottles were weighed (in grams) every 24 hr and their positions were switched to prevent side bias. On a given day, the weight of an individual bottle was subtracted from its weight from the previous day, allowing for the determination of grams (g) of liquid drank per day. Home cage bedding was changed weekly, as were the 50‐ml tubes. During the experiment, an empty cage with water and EtOH tubes was maintained as a control for evaporative loss; the volume lost was subtracted from all experimental calculations for that day. Body weights (g) of all subjects were also measured every 24 hr. For practical reasons, space limitations, males and females were run in two separate cohorts.

### 2.3 Forced swim test

The FST was used as a physical stressor and was administered between 1,500 and 1,700 hr (during their dark phase) each day for three consecutive days. During the FST, mice were maintained on the two‐bottle choice paradigm, that is, water and 11% EtOH. The only time that ethanol was not available to the experimental animals was during the FST. Mice were placed into a 19‐cm diameter Plexiglas tank filled to 24 cm with 24°C water for 10 min, as previously described by H. K. Caldwell, Dike, Stevenson, Storck, and Young (2010). Animals were videotaped and the test timed using a stopwatch. The duration of time struggling (swimming) versus. immobility was scored and calculated using Observer® (Noldus Technologies, Leesburg, VA), a behavioral coding software program. During scoring, minutes 2 through 6 were scored for each animal (total of 4 min), of which a 5‐s sampling method was utilized which categorized a mouse's behavior as swim or float. A swim behavior was defined as all four paws or the back two paws moving to propel mouse. A float behavior was defined as no paws moving or back two paws moving.

### 2.4 Taste preference test

To control for possible genotypic differences in taste preference, which can directly impact alcohol consumption (Bachmanov et al., 2003), mice were tested for their taste preferences for saccharin (sweet taste) and quinine (bitter taste). These tests were performed following the EtOH consumption experiment. All animals were presented with a choice between a tastant (saccharin or quinine) and water, each for four days in the following order: 0.033% saccharin, 0.06% saccharin, 0.015 mM quinine, and 0.06 mM quinine. Bottles were weighed every 24 hr, and the volume consumed of each tastant was calculated. During this phase of testing, one male was removed due to deteriorating health. In addition, one male and one female were removed due to sinking during FST.

### 2.5 Statistical analysis

For all statistical analysis, genotypes were compared within sex due to the fact that males and females were run at different times. Thus, only qualitative, rather than quantitative statements can be made regarding any possible sex differences. EtOH intake is expressed as grams of EtOH per kilogram of body weight per day (g/kg/day), taking into account EtOH density (0.79 g/ml) and EtOH concentration change. Percent EtOH preference is expressed as grams of EtOH solution/grams of total fluid consumed. Water and total fluid intake are expressed as grams of fluid per kilogram of body weight per day (g/kg/day). Ethanol intake, ethanol preference, water intake, and total fluid intake were then averaged across concentrations. For the FST, percent of time struggling was calculated and compared between genotypes, across the days of testing. Quinine/Saccharin intakes were averaged across concentrations to account for day‐to‐day variability in drinking patterns. Within each sex, EtOH intake, percent EtOH preference, water intake, and total fluid intake were analyzed using a repeated measure analysis of variance (ANOVA) with concentration as the within‐subject measure and genotype as the between‐subject measure. For this analysis, all 14 days post‐11% consumption were averaged. EtOH intake during the FST was analyzed using a repeated measure ANOVA with day as the within‐subject measure and genotype as the between‐subject measure. If there were any significant differences, a one‐way ANOVA was run to determine where there were any genotypic differences within a given concentration or day. A one‐way ANOVA analysis was also used to evaluate genotypic differences in the 7 days pre‐11% and the first 7 days in post‐11% ethanol consumption. Taste preference was analyzed using a two‐way repeated measures ANOVA with tastant and concentration as the within‐subject measures and genotype as the between‐subject measure. When appropriate, a Tukey *post hoc* analysis was performed. All data were verified for the appropriate assumptions prior to statistical analysis using the SPSS software (IBM, Armonk, NY). A result was considered statistically significant if *p* < .05.

## 3 RESULTS

### 3.1 Female, but not male, Oxtr −/− mice have increased voluntary EtOH consumption, both pre‐ and poststress

**FIGURE 1:**
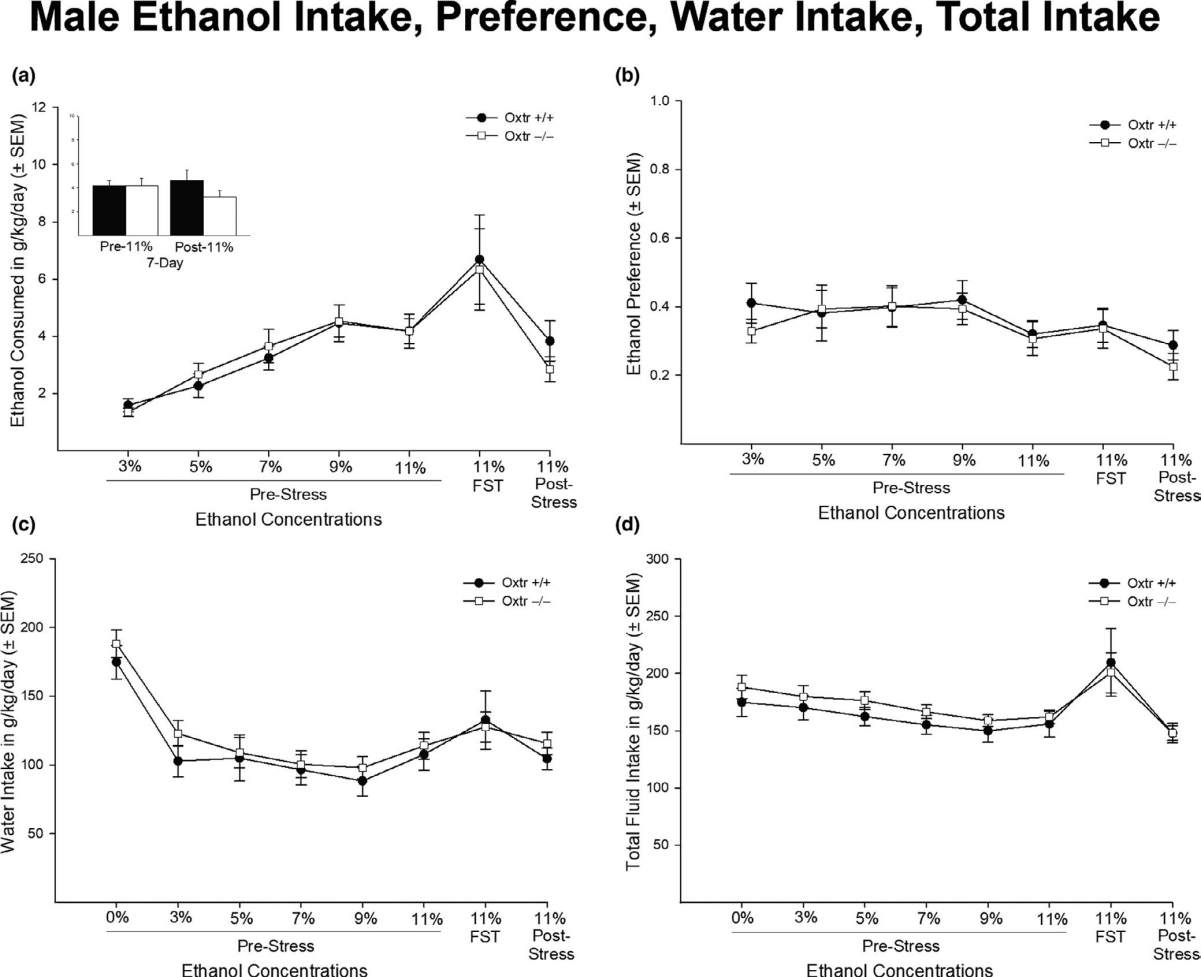
Ethanol consumption, ethanol preference, water intake, and total fluid intake in male Oxtr +/+ and −/− mice (*n* = 6 and *n* = 11, respectively*)*. Males showed no significant genotypic differences in ethanol intake consumed in grams per kilograms per day (g/kg/day) (a), ethanol preference (b), water intake shown in g/kg/day (c), and total fluid intake shown in g/kg/day (d) (*p* > .05). Males also showed no genotypic differences in the 7‐day pre‐11% intake and 7‐day post‐11% intake (*p* > .05). Percent of ethanol was calculated as grams of ethanol intake divided by total grams of fluid intake

In males, there was no statistically significant interaction between genotype and concentration in measures of EtOH intake and EtOH preference [*F* (1,16) = 0.311, *p* = .930, *F* (1,16) = 0.352, *p* = .907, respectively] (Figure 1a and b). There was, however, a main effect of concentration in both EtOH intake and preference [*F* (1,16) = 13.525, *p* < .01 and *F* (1,16) = 3.467, *p* = .004, respectively]. Figure 1a shows a 7‐day pre‐11% and 7‐day post‐11% EtOH consumption with no significant difference between genotype in either pre‐ or post‐EtOH consumption [*F* (1,16) = 0.000, *p* = .998 and *F* (1,16) = 2.050, *p* = .171, respectively]. In addition, there was no statistically significant interaction between genotype and concentration in measures of total water intake and total fluid intake [*F* (1,16) = 0.371, *p* = .918 and *F* (1,16) = 0.463, *p* = .859, respectively] (Figure 1c and d). Similar to EtOH intake and preference, there was a main effect of concentration in both measures of water intake and total fluid intake [*F* (1,16) = 20.405, *p* < .01 and *F* (1,16) = 10.060, *p* < .01, respectively].

**FIGURE 2:**
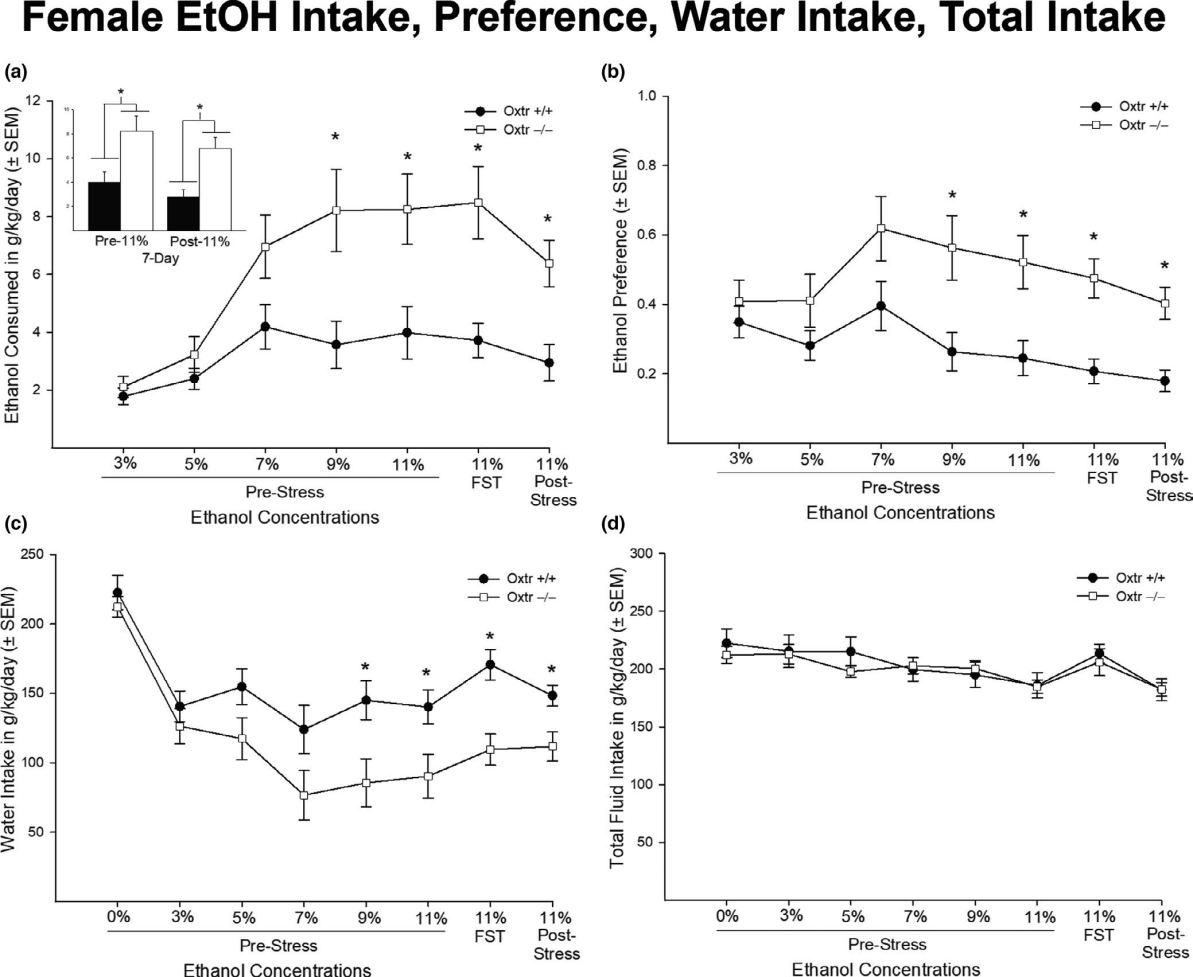
Ethanol consumption, ethanol preference, water intake, and total fluid intake in female Oxtr +/+ and −/− mice (*n* = 8 and *n* = 10, respectively). Females showed significant genotypic differences in ethanol consumption (g/kg/day) (a), ethanol preference (b), and water intake (g/kg/day) (c). Specifically, Oxtr −/− mice showed an increase ethanol intake and preference from 9% ethanol to 11% poststress ethanol compared to their wild‐type counterparts (*p* < .05). Similarly, Oxtr −/− mice showed a significant genotypic difference in the 7‐day pre‐11% intake and 7‐day post‐11% intake (*p* < .05). Conversely, Oxtr +/+ mice drank significantly more water compared to Oxtr −/− mice (*p* < .05). However, there was no significant genotypic difference in total fluid intake (g/kg/day) (d) (*p* > .05)

In females, there was a statistically significant interaction between genotype and concentration in measures of EtOH intake and preference [*F* (1,17) = 6.557, *p* < .01 and *F* (1,17) = 3.019, *p* = .009, respectively] (Figure 2a and b). *Post hoc* analysis identified significant genotypic differences with female Oxtr −/− mice having increased EtOH intake compared to their wild‐type counterpart. Similar to males, females showed a main effect of concentration in measures of EtOH intake and preference [*F* (1,17) = 23.314, *p* < .01 and *F* (1,17) = 7.420, *p* < .01, respectively]. Figure 2a shows a 7‐day pre‐11% and 7‐day post‐11% EtOH consumption with a significant difference between genotype in pre‐ [*F* (1,17) = 7.595, *p* = .013] and post‐EtOH consumption [*F* (1,17) = 12.119, *p* = .003]. In addition, females showed a statistically significant interaction between genotype and concentration in measures of water intake with Oxtr −/− mice drinking less water compared to Oxtr +/+ mice [*F* (1,17) = 2.524, *p* = .019] (Figure 2c). There were no statistically significant genotypic differences in total fluid intake [*F* (1,17) = 1.078, *p* = .382] (Figure 2d). However, there was a main effect of concentration in total fluid intake [*F* (1,17) = 12.386, *p* < .01], as well as water intake [*F* (1,17) = 34.995, *p* < .01].

### 3.2 Stress did not result in genotypic‐ or sex‐specific increases in alcohol consumption

**FIGURE 3:**
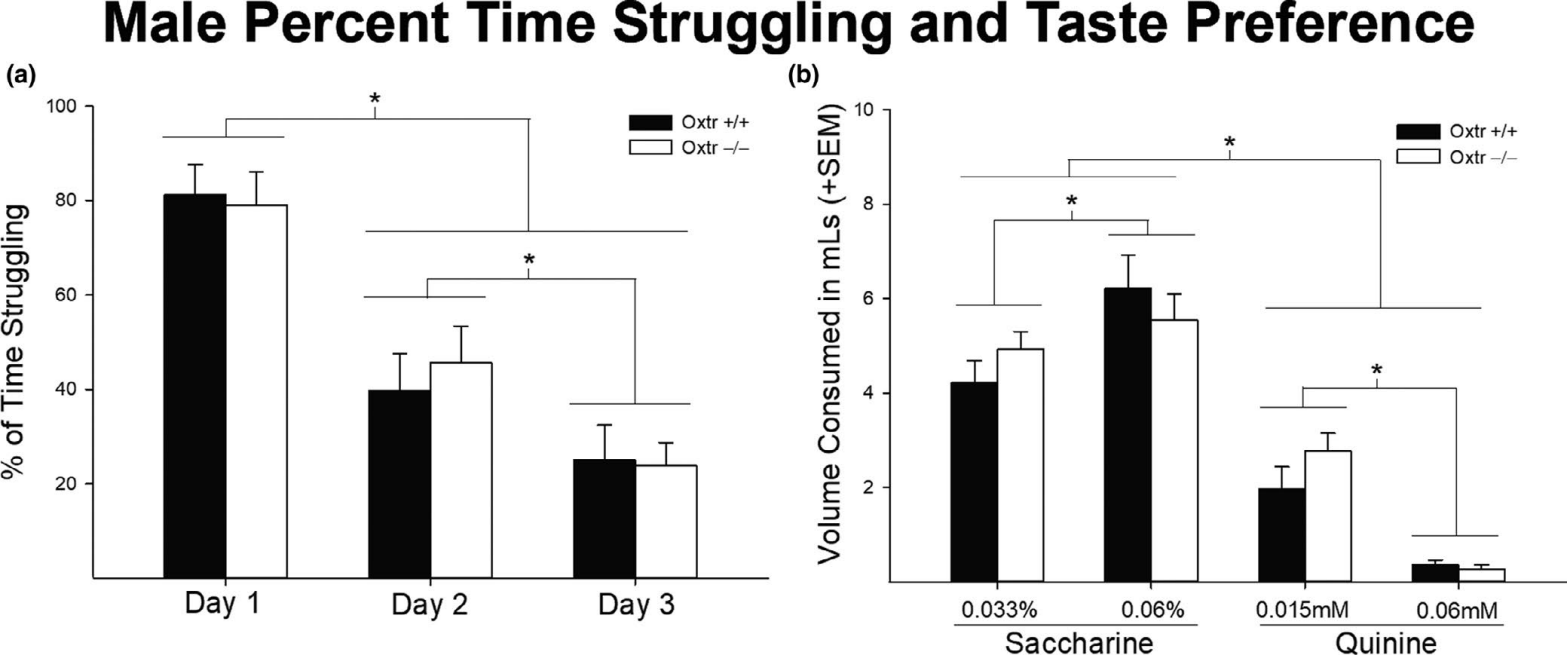
Forced swim stressor and taste preference in male Oxtr +/+ and −/− mice (*n* = 6, *n* = 11). The forced swim analysis showed no significant genotypic differences in percent of time struggling (*p* > .05), and however, there was a significant effect of days (a). Males decreased their percent time struggling throughout days of testing (*p* < .05). In taste preference, there were no statistically significant genotypic differences (*p* > .05) (b). However, there was a significant effect of tastant and concentration, where males preferred saccharin over quinine, high concentration of saccharin over low concentration of saccharin, and lower concentration of quinine over higher concentration of quinine (*p* < .05)

**FIGURE 4:**
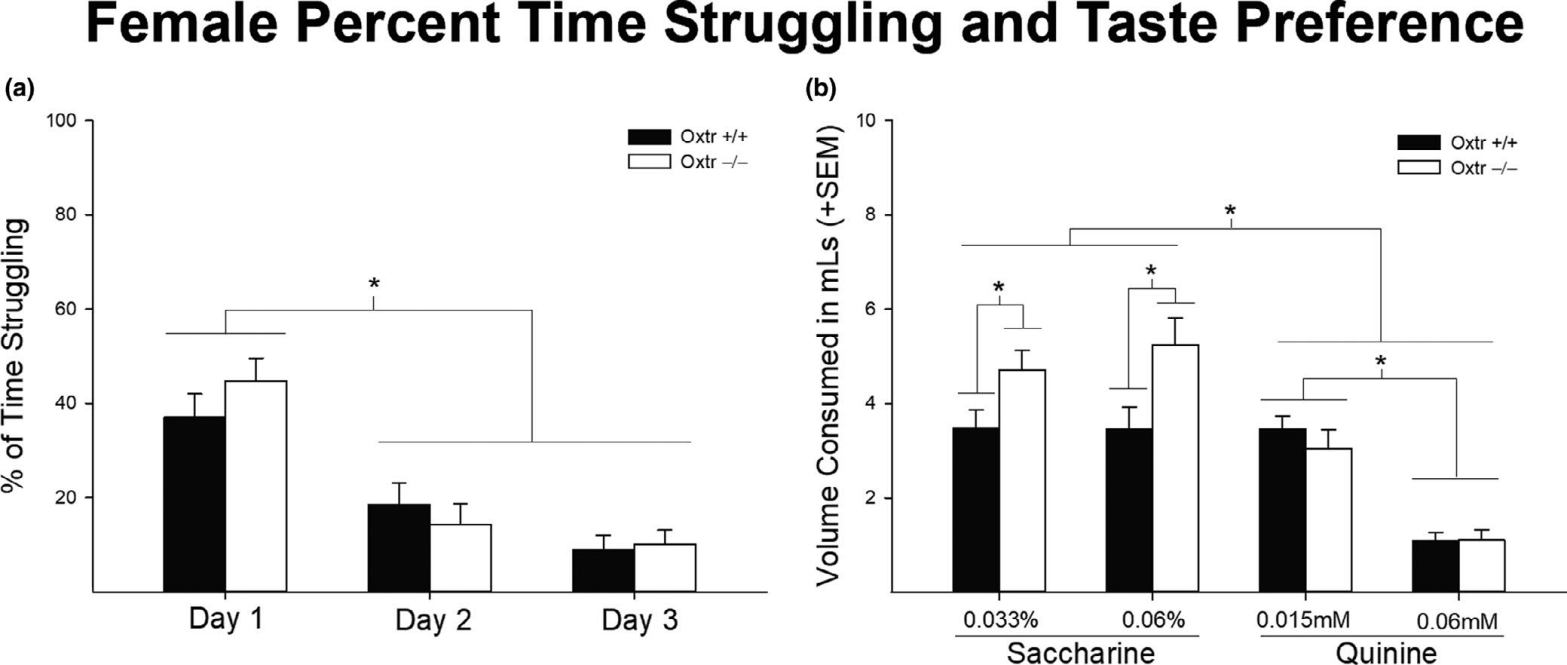
Forced swim stressor and taste preference in female Oxtr +/+ and −/− mice (*n* = 8, *n* = 10). There were no significant genotypic differences in percent of time struggling (*p* > .05), and however, there was a significant effect of days (a). Females decreased their percent time struggling throughout days of testing (*p* < .05). In taste preference, there was a statistically significant genotypic difference in saccharine where Oxtr −/− females preferred more saccharine compared to wild‐type counterparts (b). In addition, females also showed a significant effect of tastant and concentration (*p* < .05). Like males, females preferred saccharin over quinine and lower concentration of quinine over higher concentration of quinine (*p* < .05)

**FIGURE 5:**
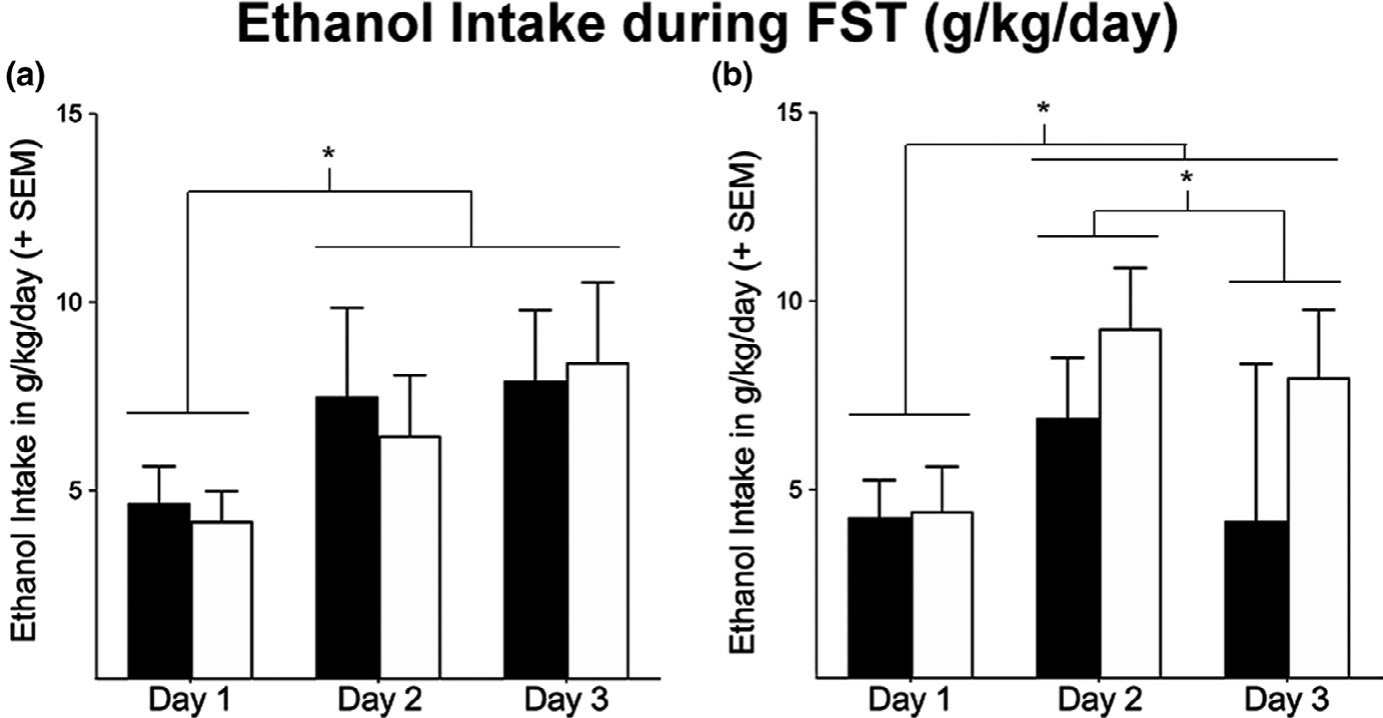
Ethanol intake in male and female Oxtr +/+ and Oxtr −/− mice (*n* = 6, *n* = 11; *n* = 8, *n* = 10, respectively) during the forced swim test. Within each sex, there were no significant genotypic differences in ethanol intake (*p* > .05). There was, however, a significant effect of days in both male (a) and female (b) mice (*p* < .05)

Neither males or females showed any statistically significant genotypic differences in percent of time struggling [*F* (1,16) = 1.080, *p* = .352 and *F* (1,17) = 1.525, *p* = .232, respectively] (Figures 3a and 4a, respectively). There was, however, a main effect of day in both male [*F* (1,16) = 114.268, *p* < .01] and female [*F* (1,17) = 46.012, *p* < .05] Oxtr −/− mice, with both sexes decreasing the percent time struggling each day. In addition, the FST stressor did not result in any genotypic differences in alcohol consumption. Neither males or females showed any statistically significant genotypic differences in ethanol intake during the three consecutive days of FST [*F* (1,16) = 0.025, *p* = .877 and *F* (1,17) = 1.422, *p* = .249, respectively]. There was also no significant interaction of days x genotype in either males or females [*F* (1,16) = 0.283, *p* = .755 and *F* (1,17) = 2.916, *p* = .068, respectively]. There was, however, a main effect of day in both male [*F* (1,16) = 6.838, *p* = .003] and female [*F* (1,17) = 11.973, *p* < .05] Oxtr −/− mice, with both sexes, in general, increasing their ethanol intake across days (Figure 5).

### 3.3 *Female Oxtr* −/−* mice have an increased preference for the sweet tastant*

In males, there was no statistically significant interaction of genotype in sweet (saccharine) or bitter (quinine) tastant consumption [*F* (1,16) = 0.301, *p* = .591] (Figure 3b). However, there was an expected main effect of tastant type, with higher volumes of saccharine being consumed compared to quinine [*F* (1,16) = 106.561, *p* < .05]. Further, there was an interaction of tastant and concentration with higher volumes of saccharine (0.06%) and quinine (0.015 mM) being consumed compared to their other respective concentrations [*F* (1,16) = 54.105, *p* < .05]. Unlike males, females showed a statistically significant interaction between genotype and tastant specifically in saccharine consumption [*F* (1,17) = 8.119, *p* = .011], with Oxtr −/− females consuming higher volumes of saccharine compared to their wild‐type controls independent of concentration (Figure 4b). Similar to males, females displayed the expected main effect of tastant [*F* (1,17) = 46.670, *p* < .01], as well as a tastant by concentration interaction [*F* (1,17) = 36.375, *p* < .01].

## 4 DISCUSSION

In this study, we have evaluated alcohol consumption in male and female Oxtr −/− mice before and after a physical stressor. We hypothesized that there would be genotypic differences in stress‐induced alcohol consumption, with both male and female Oxtr −/− mice having increased alcohol intake and preference compared to their wild‐type control littermates following a physical stressor. Although, we predicted that these effects might be greater in female Oxtr −/− mice. Interestingly, starting with administration of 9% EtOH and throughout the remainder of the experiment, only female Oxtr −/− mice had an increase in their ethanol consumption relative to controls. It is of note that female wild type and −/− littermates had similar EtOH intake levels until the administration of 7% EtOH. From the 7% EtOH and on, wild‐type females appear to decrease their EtOH intake while Oxtr −/− females increased their ethanol intake, and however, there were no genotypic differences in EtOH intake on the days of the FST stressor. These data suggest that in females, a functional Oxtr has protective effects against the consumption of higher concentrations of EtOH.

Conversely, in males, we found no genotypic differences in alcohol consumption and preference either pre‐ or poststress. Though, males appear to have found the FST fairly aversive, perhaps even more so than females, as the males spent an average of 80% of their time struggling on day one of testing compared to an average of 40% in females. We did observe the expected increase in EtOH intake during the FST, which did not have any long‐term effects on alcohol consumption. This is particularly interesting in light of the fact that males spent much more time struggling during the forced swim stressor than females. As previous studies have suggested, it may be that sex differences in Oxt are directly regulating sex‐specific behaviors [for review see Caldwell (2018), Dumais and Veenema (2016)], in this case alcohol consumption. Given the results reported herein, it is reasonable to speculate that, in females, loss of oxytocinergic receptor function increases the vulnerability of females to develop an AUD, as observed in clinical populations (Vaht et al., 2016).

Since it is known that, compared to males, females tend to have higher blood alcohol levels after drinking, differ in their alcohol metabolism, and have greater neurotoxic effects (Anker & Carroll, 2011), it is plausible that some of our observed effects in females are due to sex‐dependent pharmacological effects of EtOH. EtOH is known to have anxiolytic, sedative, locomotor, and reinforcing effects and given that there are a limited number of studies that have evaluated alcohol intake in Oxtr −/− mice, it is difficult to say whether our results are due to genetic disruption alone or in tandem with the physiological effects of ethanol. For example, it may take less alcohol consumption for male Oxtr −/− mice to feel the sedative and/or rewarding effects of alcohol compared to females. Female Oxtr −/− mice may consume a lot more alcohol just to feel its rewarding properties. While beyond what is presented here, it would be useful to determine whether there are genotypic differences in how alcohol is metabolized, this could be achieved by measuring their blood alcohol concentration.

Beyond these possible sex and/or genotypic differences in the effects of alcohol, it is also important to consider that the Oxt system closely interacts with the dopaminergic reward system. This system, while important to aspects of motivated social behaviors, is also known to be important in addictive states (Bowen & Neumann, 2017; Caldwell & Albers, 2016). Further, the Oxtr is expressed in brain regions known to be part of the reward neural circuitry (Caldwell & Albers, 2016; Peris et al., 2017). Oxt projections from the PVN synapse on dopaminergic neurons of the NAcc (Knobloch & Grinevich, 2014) and the Oxtr is expressed on dopamine neurons, specifically those projecting from the VTA to the NAcc and medial prefrontal cortex (Peris et al., 2017). A recent study has shown that ICV Oxt injection blocks dopamine release induced by ethanol administration within the NAcc, which results in reduced ethanol seeking behavior (Peters, Bowen, Bohrer, McGregor, & Neumann, 2017). Thus, it is very likely that our female Oxtr −/− mice are missing the neurochemical “brake” for dopamine release, resulting in an overactive reward pathway. If this is the case, it would explain why female Oxtr −/− mice consume more ethanol than wild‐type controls. It does not, however, explain why there was no genotypic difference in EtOH consumption in our male mice. Moving forward, work critically examining the interaction of the Oxt and dopaminergic systems in female and male Oxtr −/− mice will be an important “next step”.

Unlike female Oxtr −/− mice, Oxtr −/− males showed no genotypic differences in alcohol preference and consumption. This could be because of an unknown compensatory mechanism that may exist in our male Oxtr −/− mice that allows for Oxt to continue to signal and buffer alcohol consumption. One option would be the arginine vasopressin (Avp) system. Oxt and Avp are evolutionarily related neuropeptides that only differ by two amino acids and have known affinities for each other's receptors (Caldwell & Albers, 2016; Neumann & Landgraf, 2012). It is possible that in males, Oxt reduces the motivational effects of alcohol through actions on one of the centrally expressed Avp receptors (Neumann & Landgraf, 2012). For instance, in genetically high alcohol‐preferring rats and alcohol‐dependent Wistar rats, the Avp 1b receptor (Avpr1b) antagonist SR 149415 reduces alcohol consumption (Edwards, Guerrero, Ghoneim, Roberts, & Koob, 2012; Zhou et al., 2011). Thus, further research is needed to test the role of Avp signaling as a protective compensatory mechanism in male Oxtr −/− mice.

Studies have shown that the Oxt system plays a major role in feeding behavior, caloric intake, and taste preference (Arletti, Benelli, & Bertolini, 1989; Lawson et al., 2015). In this study, female Oxtr −/− mice have an increased preference for saccharine as compared to water. This difference is not observed in male Oxtr −/− mice, which is consistent with other work performed in male Oxtr −/− mice (Lee et al., 2008). Additionally, the female data presented here also consistent with previous work demonstrating that experimentally naive Oxt −/− females have a robust initial and sustained preference for sucrose and saccharin compared to their wild‐type counterparts (Amico, Vollmer, Cai, Miedlar, & Rinaman, 2005; Billings, Spero, Vollmer, & Amico, 2006; Sclafani, Rinaman, Vollmer, & Amico, 2007). Thus, we can conclude that Oxt signaling pathway is able to modulate the intake of sweet solutions in mice. Perhaps this preference for sweet solutions is why female Oxtr −/− mice consumed more alcohol compared to controls, as low concentrations of alcohol are known to have a sweeter taste compared to higher concentrations (Crabbe, Phillips, & Belknap, 2010). Though, it is important to acknowledge that differences in taste preference in females, and lack thereof in males, could also be due to carry‐over effects from the FST.

At this time, current treatments for AUD involve a combination of psychological, social, and pharmacotherapeutic interventions, with the best pharmacological therapies reducing the rate of relapse and cravings, and decreasing the frequency of drinking following relapse (Kiefer & Mann, 2005). That being said, there is always an interest in developing more efficacious pharmacological treatments. The challenge of course is that it is likely that many neurochemical changes are contributing to AUD, and however, Oxt has shown preclinically and clinically to have positive beneficial effects. In humans, there is evidence that patients diagnosed with an AUD and treated with intranasal Oxt have reduced cravings (Lee, Rohn, Tanda, & Leggio, 2016) and alcohol withdrawal symptoms (Pedersen et al., 2013). Nevertheless, to understand the benefits of Oxt as a therapeutic, it is especially important to study the role of the Oxt system in alcohol consumption not only in males, but also in females as results can differ as a function of an individual's sex.

It is a societal misconception that alcoholism or alcohol‐related problems are male‐specific disorders. Interestingly, sex‐specific gender norms are shifting and this male‐leaning prevalence gap is diminishing (Keyes, Martins, Blanco, & Hasin, 2010), which only increases the need for female inclusion in drug abuse and addiction research. The preliminary results in this study are the first to address this gap, and however, further research is necessary to better understand the Oxt system and its role in alcohol consumption. Undoubtedly, the use of genetically modified animals is a great tool to tease apart the possible mechanism between increased alcohol consumption and disruption of the Oxt system, focusing on sex‐specific differences. Future studies will include measuring the rewarding properties of alcohol in both males and females, as well as looking for possible brain areas that may better explain the neuro‐connectivity or lack thereof due to loss of oxytocinergic function.

## 5 CONCLUSIONS

Our study is the first to investigate alcohol consumption in both male and female Oxtr −/− mice. Our data suggest that impairments in the functioning of the Oxt system may make females, but not males, more vulnerable to increased EtOH consumption. While it is clear that additional research is needed to better understand the contributions of the Oxt system to the vulnerability associated with alcohol preference and consumption, our data also underscore the importance of considering sex as a biological variable in these types of studies. These data also support previous work which suggest that the Oxt system may be a possible pharmacological target to aid in the prevention of addictive behaviors, such as alcohol abuse disorders.

## CONFLICT OF INTEREST

The authors of the manuscript have no conflicts of interest to declare.

## AUTHORS’ CONTRIBUTIONS

Author contributions were as follows: Karla M. Rodriguez participated in experiment planning, animal husbandry, experimental acquisition of data, data analysis, writing the manuscript, and creating figures, Brittany L. Smith participated in experiment planning and writing the manuscript, and Heather K. Caldwell participated in experiment planning, data analysis, and writing the manuscript.

### Peer Review

The peer review history for this article is available at https://publons.com/publon/10.1002/brb3.1749.

## Data Availability

The data that support the findings of this study are available from the corresponding author upon reasonable request.
